# Radiovaccination Strategy for Cancer Treatment Integrating Photodynamic Therapy-Generated Vaccines with Radiotherapy

**DOI:** 10.3390/ijms232012263

**Published:** 2022-10-14

**Authors:** Mladen Korbelik

**Affiliations:** BC Cancer, Vancouver, BC V5Z 1L3, Canada; mkorbelik@bccrc.ca; Tel.: +1-604-675-8084

**Keywords:** radiovaccination, cancer vaccine, photodynamic therapy

## Abstract

Therapeutic cancer vaccines have become firmly established as a reliable and proficient form of tumor immunotherapy. They represent a promising approach for substantial advancements in the successful treatment of malignant diseases. One attractive vaccine strategy is using, as the vaccine material, the whole tumor cells treated ex vivo by rapid tumor ablation therapies that instigate stress signaling responses culminating in immunogenic cell death (ICD). One such treatment is photodynamic therapy (PDT). The underlying mechanisms and critical elements responsible for the potency of these vaccines are discussed in this review. Radiotherapy has emerged as a suitable component for the combined therapy protocols with the vaccines. Arguments and prospects for optimizing tumor control using a radiovaccination strategy involving X-ray irradiation plus PDT vaccines are presented, together with the findings supporting its validity.

## 1. Introduction

Therapeutic cancer vaccine development remains an important medical need for the substantial advancements of an effective treatment of malignant diseases [1,2]. There are currently hundreds of such vaccine preparations under development that have entered clinical trials [3]. The aim of using vaccines is to provide means for active immunization to hold in check and destroy selectively tumor cells in a systemic fashion, thus ensuring the eradication of metastatic deposits and averting tumor recurrence [4]. While such vaccines have great potential for tumor immunotherapy, their clinical results as a standalone therapy are still dominated by negative outcomes in phase III trials [5,6].

Principal therapeutic cancer vaccine formats are whole-tumor-cell vaccines (autologous and allogenic), dendritic cell vaccines, peptide vaccines, oncoviral or microbial vector vaccines, nucleic acid vaccines (mRNA- or DNA-based), and in situ vaccines [2,4,7]. The latter refers to any approach where a direct intervention aimed at the tumor site exploits available local antigens to induce antitumor immune response [8]. The methodology based on whole-tumor-cell vaccination is in the forefront of this field as it secures access to all the antigens of tumor cell without any selection/bias [4].

While vaccines may effectively secure production of cancer cell killing T cells, a formidable task remains in ensuring the timely accumulation of these effectors in sufficient numbers in the tumor and adequately retaining their potency for overcoming completely tumor defenses [4]. Moreover, there is a difficulty in inducing an effective antitumor immune response in compromised immune system of cancer patients [5]. Thus, for the treatment of many tumors, combining vaccines with other tumor-targeting therapies appears indispensable, particularly for securing the required duration of potency and effectiveness of the antitumor immune activity [9]. The present study describes the applicability of using radiovaccination strategy (combining therapeutic cancer vaccines with radiotherapy) for optimizing the efficacy of photodynamic-therapy-generated vaccines.

## 2. Photodynamic Therapy-(PDT)

Therapeutic intervention known as PDT is clinically established for treatment of various cancers and also non-oncological indications, and is actively being developed for novel applications as well as for improved performance based on novel photosensitizers and/or advanced nanotechnology [10,11]. Among its advantages compared with other approved clinical modalities are function-preserving quality, absence of cumulative toxicity, minimally invasive character and excellent cosmetic effect [10,12]. The procedure for PDT is based on the use of nontoxic photosensitizing drug (photosensitizer) administered to patients systemically or locally followed by local nonthermal irradiation of targeted lesion with a specific wavelength of light matching the photosensitizer absorption profile. The photosensitizers are usually porphyrins, chlorins, or related aromatic compounds capable of capturing visible light energy and directing it into type II Photochemical reactions [10,13]. In this reaction, the photosensitizer in its excited state interacts with molecular oxygen producing reactive oxygen species (ROS) dominated by singlet oxygen. These cytotoxic species oxidize key cellular macromolecules leading eventually to tumor cell death [13]. By its nature, PDT belongs to the class of direct tumor ablation therapies that are performed by a direct local application of energy and/or chemical agent to the targeted tumor aiming for its rapid in situ destruction [14]. In addition to nonthermal illumination used for PDT, technologies used for diverse tumor ablation therapies include various forms of thermal energy delivery and electric field exposure [15,16].

Destruction and eradication of tumors by PDT treatment has been attributed to three distinct but inter-related mechanisms [10,13]. The immediate is the direct killing of malignant cells by PDT-generated ROS with cytotoxic impact on tumor cells [17]. Next is the shutdown of the tumor vascular structure caused by the photochemical damage to the endothelial layer in tumor blood vessels leading to blood flow reduction with consequently impaired supply of oxygen and nutrients resulting in tissue starvation [11]. The third component is the induced immune reaction directed against PDT-treated tumor [11,18,19].

## 3. PDT-Induced Anti-Tumor Immune Response

The capacity to elicit a strong immune rejection of treated tumors PDT shares with other rapid tumor ablating modalities [11]. The basis of this effect is in the exacted stress response in targeted cancer cells. With PDT, the imbalance between the emergence of generated ROS and the ability of afflicted cells to secure their prompt detoxification or repair the resulting damage is regarded as the inflicted oxidative stress [20]. Oxidative stress is associated not only with the appearance of oxidative damage to cellular proteins and lipids, but also with disturbances in the normal cellular redox state or reduced oxygen tensions. Primary stressors in PDT-treated cells are misfolded proteins accumulated in elevated levels particularly in the endoplasmic reticulum (ER), which is the site of folding and maturation of transmembrane, secretory, and other proteins in the cell [21].

The infliction of oxidative stress in PDT-treated cells causes a threat of proteostasis impairment, which prompts the engagement cellular stress signaling networks [22]. The action of these signaling networks determines the fate/survival of treated cells and the outcome of tumor PDT [23]. This homeostatic, evolutionary well-conserved canonic universal stress protection framework is operated by the elaborate harmonized system of interlinked intracellular signal transduction pathways [24]. The activity of stress signaling networks mitigates the stress challenges either by improving the resistance to this insult or by reducing the stress impact with increased tolerance [25]. This is accomplished by enhancing the clearance of produced primary stressor molecules or by limiting the stress damage (for example, by improving protein folding capacity), respectively. The corresponding response to PDT-triggered stress trauma is engaged, at least initially, to enable the cells to defend and recover from the insult, but if the damaging insult persists and remains unresolved then the signaling cascades switch towards self-destructive programs promoting cell death, disposal of damaged material and cell corpses, or elimination by immune rejection [22].

Stress response signaling interacts at multiple points with the immune signal transduction activities [23]. This includes crosstalk with inflammatory signaling, which controls key regulators such as nuclear transcription factor NFκB and Toll-like receptor expression, signals regulating innate and adaptive immune activity, as well as the activity of regulatory immune cells [22,26]. Additionally, integrated is the control of signaling cascade responsible for the release of alarmins and expression of damage-associated molecular patterns (DAMPs), as well as the induction of immunogenic cell death (ICD) [11,23]. Another development promoting the anti-tumor immune response progression is the abundant availability of exposed tumor antigens made accessible upon the execution of programmed cell death pathways, with ensuing removal and processing of dead tumor cell material mediated by phagocytes endowed with antigen presentation activity [27]. A particularly determining development is the stressed cells becoming highly immunogenic due to the stress-response-induced expression of cryptic tumor antigens normally hidden within untranslated regions of tumor cell RNA [28]. This is sanctioned by stress signaling-triggered accrual of alternate initiation factors capable of translating normally unreadable RNA regions [29]. The exposed neoantigens render stressed cells strongly immunogenic because they are not protected by immunotolerance mechanisms. 

## 4. PDT-Generated Cancer Vaccines

Therapeutic cancer vaccine generated by PDT has been in the focus of our research for the past two decades. We have closely followed the initial work of Gollnick, Henderson and coworkers first describing basic characteristics of PDT vaccines [30,31]. This first paper has shown that the lysate of in vitro PDT-treated mouse tumor cells can serve as a prophylactic vaccine as its injection protected mice against subsequent challenge with the same tumor but not against a miss-matched (different tumor) [30]. The PDT vaccine protocol developed in our laboratory was primarily established for the therapeutic use and was based on whole tumor cells [31]. In this case, the vaccine material consisted of autologous cancer cells undergoing ICD due to their treatment by PDT in vitro [32]. Using localized injection of ex vivo PDT-treated tumor cells avoids some of the risks (side-effects) associated with systemic (in situ) PDT treatment such as skin photosensitivity induced by some photosensitizers [10].

Further research by various investigators produced a number of important findings. It has become clear that the effectiveness of these vaccines is not dependent on a particular photosensitizer as a variety of them have been successfully tested [33]. Optimal PDT dose and tumor cell number per vaccine dose need to be identified for different PDT vaccine protocols [34]. An important aspect is that minced surgically removed tumor tissue can be used for the vaccine without obligatory establishment of cancer cell cultures [34].

One of the critical beneficial mechanisms for enhancing the effectiveness of the vaccines is chemoattracting APCs into the vaccination site, and such activity was demonstrated with PDT vaccines [33].

Convincing evidence has accumulated verifying that the PDT vaccine effect is attained by a targeted tumor-specific immune rejection executed by cytotoxic T cell action. This includes:

- Resistance against re-challenge with a vaccine-cured tumor;
- Failure of protecting against different mismatched tumors;
- Effective control of tumors growing distantly from the vaccination site;
- Mobilization of dendritic cells (DCs) to vaccination area and their functional maturation;
- Induction of vaccinated tumor-specific interferon-γ-secreting T cells with enhanced selective tumoricidal activity;
- Appearance post-vaccination of elevated numbers of degranulating CD8^+^ T cells in regressing lesions, but not in the poor responders;
- Absence of the vaccine effect in cytotoxic T cell-depleted hosts [33].

A key factor for the efficacy of PDT vaccines is the expression in the vaccine cells of PDT-induced ICD and other changes associated with the cell death as well as the expression of DAMPs and other molecular/biological changes in these cells [35]. Another important discovery is that the PDT vaccine treatment induces a form of acute phase response reaction and hormonal axis activation in the host that influence the activity of genes at distant sites including liver and spleen [36].

Two especially critical elements in the mechanism of antitumor immune response development elicited by whole-tumor-cell-based PDT vaccines are the induced stress signaling mediated death programs and the mobilization of efferocytosis (cell disposal) pathways instrumental for the accrual and presentation of tumor antigens contained in the vaccine material [32,37]. This enables a potent enhancement of the vaccine efficacy by targeted modulation of death programs including apoptosis, necrosis and autophagy [32]. On the other hand, this offers a strategy of influencing phagocytic receptors functioning on patient’s antigen-presenting cells (APCs) [37]. Thus, the therapeutic impact of PDT vaccines was shown to become abolished by blocking scavenging receptors such as LOX-1, while it can be significantly enhanced by blocking immune inhibitory receptor FcγRIIB [37].

Among important contributions to the knowledge about PDT-generated vaccines and their optimization is the establishment of the vaccines based on DCs pulsed with PDT-treated tumor cells [38], or their lysate [39]. More details of PDT vaccines for cancer are summarized in Table 1.

Immunosuppressive elements maintained and controlled by immunoregulatory cell populations have emerged as obligatory targets needed to be neutralized for the success of not only cancer vaccines but also most of different types of cancer therapy [44]. We have reported that two dominant immunoregulatory populations, lymphoid Tregs and myeloid-derived suppressor cells (MDSCs), have a critical negative impact on therapy outcome with PDT vaccines [45,46]. Moreover, reducing their numbers and/or blocking their activity were demonstrated to substantially improve the effectiveness of PDT vaccines [45,46,47,48].

## 5. Cancer Radiotherapy Combined with Vaccines

Since cancer vaccines appear in many cases insufficient for securing a positive clinical outcome when used as standalone therapy [49], it is increasingly recognized that their utility could be better established by combining them with either traditional modalities such as radiation, chemotherapy and surgery or with novel regimens aimed at modulating tumor microenvironment [4,50,51]. The same pertains also to PDT vaccines [33].

Radiotherapy is an established standard of care for many malignancies; therefore, it features as one of the prominent candidates for enhancing responses when combined with cancer vaccines [52]. Increased interest in recent years in developing protocols for combining radiotherapy with cancer vaccines presents actually a paradigm shift [53], because radiotherapy was traditionally considered immunosuppressive [54]. Indeed, radiotherapy can reduce the numbers of tumor-infiltrating immune effector cells during the irradiation treatment regimens [55] and there were reports of decreased nonspecific immune system responses that remained suppressed for months after radiation [54,56]. However, such consequences can be minimized by avoiding exposure of multiple lymph node chains to radiation. Among other effects of radiotherapy that could decrease immunogenic responses are its reported induced upregulation of immune regulatory cytokine TGF-β and transcriptional regulator HIF-1α [57,58].

On the other hand, local radiation exposure of the tumor site was sometimes found to result in the reduction in non-irradiated distant metastases evidently mediated by the immune system; this phenomenon is known as the abscopal effect [59]. Indeed, numerous studies are coming to prominence that have demonstrated immunogenic properties of radiotherapy:

- Exposure of tumors to radiation was shown to alter the phenotype of tumor cells rending them more susceptible to immune cell killing; the underlying changes include increased expression of MHC class I molecules, death receptors, and surface adhesion molecules [60,61].
- Surface calreticulin expression on tumor cells induced by radiation treatment was also connected to the observed enhanced T cell killing [62].
- Increased expression of costimulatory molecules for T cells, including OX-40L and 4-1BBL, in irradiated tumor cells has also been reported and suggested to promote antitumor immune interaction [63]. Positive effects from elevated expression of these costimulatory molecules could also result from their negative impact on immunosuppression mediated by tumor-mobilized Tregs and other immunoregulatory cells [64,65].
- Radiation treatment was found to result in the production of chemoattractant factors that increase the migration of T cells into tumors [66]. Here, radiotherapy acts as a vascular remodeling agent stimulating the recruitment of inflammatory and active immune effector cells [67].
- Radiotherapy can alter (sometimes reduce) viability of both Tregs and MDSC populations in the tumor microenvironment [68].

The molecular mechanisms associated with radiation-triggered immunogenic modulation include alterations in the expression of antiapoptotic/survival and/or immune response genes linked to immunogenic cell death (ICD) [69].

Encouraging results of preclinical and clinical studies of combined radiotherapy and therapeutic cancer vaccines are illustrated by the following examples:

- Synergy of radiotherapy and cancer vaccine based on B subunit of the Shiga toxin (STxB) coupled with HPV16 E7 oncoprotein was demonstrated on a pre-clinical mouse model of head and neck tumor [70]. The non-replicative vector STxB targets dendritic cells, and when coupled to various tumor antigens elicits a strong specific CTL-based antitumor immune response [71]. The therapeutic efficacy against head and neck tumor of the vaccine was found to be strongly enhanced by local radiation. This was linked to the induction of a more potent antitumor immune response in the combined therapy group that could at least in part be attributed to increased tumor vascular permeability promoting migration of immune effector cells into the tumor [70].
- Combining a recombinant cancer vaccine with standard radiotherapy in patients with localized prostate cancer was examined in phase II clinical trial [72]. The used poxviral vaccine encoding prostate-specific antigen (PSA) effectively induced a PSA-specific T cell response when combined with radiotherapy, and this procedure was safe. Such a response was not detectable in the radiotherapy-only arm.
- A clinical trial evaluated responses to autologous DC-based vaccine in combination with conformal radiotherapy from 40 patients with recurrent metastatic or locally advanced tumors of the pancreas, lung, esophagus, uterus, or head and neck [73]. For the vaccine, matured DCs pulsed with autologous tumor cell lysates or tumor-specific peptides were administered every other week after radiotherapy, up to seven times. A response rate of 61% was documented for patients receiving full-dose radiotherapy. In overall, the results suggested that the combination of DC-based vaccine and RT induces evaluable clinical responses [64,73].

## 6. Radiovaccination with PDT Vaccines

As a promising cancer vaccine modality, PDT-generated therapeutic vaccines are also attractive candidates for the combination regimens involving conventional radiotherapy. Radiotherapy is usually performed before the vaccine delivery with the intent of avoiding destruction by ionizing radiation of activated immune effector cells massively mobilized to invade the vaccinated tumor.

**Figure 1:**
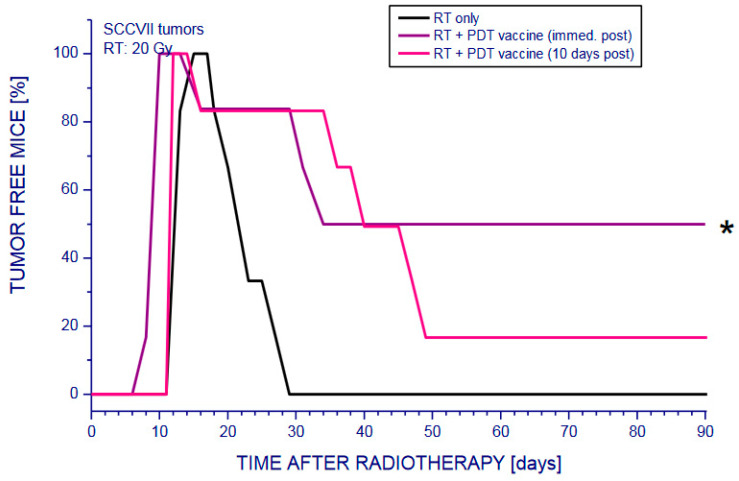
The response of mouse SCCVII tumors to PDT vaccines combined with radiotherapy. Mice bearing SCCVII tumors received a peritumoral injection of SCCVII cells that were treated in vitro by ce6-PDT followed by 16 h post-incubation in culture, as described in detail elsewhere [47]. For radiotherapy, the tumors were exposed to X-rays (20 Gy) with mice immobilized in lead holders. The mice were then monitored 90 days for signs of tumor regrowth and those remaining impalpable after this interval were considered cured. Each treatment group consisted of 6 mice. * Statistically significant difference (*p* < 0.05) compared with other treatment groups. The result for PDT vaccine alone treatment was presented elsewhere [46].

To investigate this combination in a pre-clinical setting, we have used an autologous whole-cell PDT vaccine protocol characterized and optimized in our laboratory through extensive investigations during the past two decades [27,32,33,34,35,36,46]. Mouse squamous cell carcinoma SCCVII, a well-recognized immunotherapy model for head and neck cancer [74] was utilized in these studies. For the vaccination of SCCVII tumor-bearing mice, 20 million SCCVII cells treated in vitro by PDT (incubation with 0.5 µg/mL of photosensitizer chlorin e6 for 30 min followed by exposure to 1 J/cm^2^ of 665 nm light) were injected peritumorally per mouse [46]. Experimental groups were radiotherapy (RT) alone, PDT vaccine alone, radiotherapy followed immediately by a single PDT vaccine treatment, and radiotherapy plus single PDT vaccine administration 10 days later. From the results, it can be seen that the chosen RT alone protocol rendered tumors impalpable only between 1–4 weeks after treatment, which was followed by visible recurrence of all tumors resulting in no permanent cures (Figure 1). A similar impact with no permanent cures was evidenced with PDT vaccine alone treatment (not shown), as presented earlier [46]. In contrast to these limited impacts of tested single modalities, the therapy outcomes were more successful with the combined treatment protocols (Figure 1). This was especially evident with PDT vaccine administered immediately after RT, which produced around 50% tumor cures. With the vaccine treatment delayed 10 days, the tumor cure rate was below 20% and was statistically not different than RT alone group. This suggests that with the vaccine given immediately after radiotherapy the interaction between the two modalities was not purely additive, but synergistic.

The nature of this interaction synergy needs additional investigation to become fully elucidated. The underlying contributing factors are probably multiple and include:

- De-bulking the tumors by RT (cytoreduction) that allows the PDT vaccine-activated immune mechanisms to engage with more easily eradicated smaller malignant deposits;
- Vascular re-modeling by RT facilitating the tumor invasion of immune effector cells mobilized by PDT vaccine [66,67];
- Induction of ICD signaling not only by PDT vaccine but also by RT allowing the presentation of a much wider range of tumor antigens/neoantigens for a much broader antitumor immune attack [69];
- Dampening the activity of immunoregulatory elements in the tumor environment by the increased expression of T cell costimulatory signals caused by RT treatment [64,66].

## 7. Conclusions

Given the encouraging supporting pre-clinical findings and improved understanding of the underlying mechanisms, as well as their clinical potential described in the present article, it is clear that the strategy of combining therapeutic PDT-generated cancer vaccines with conventional radiotherapy is worth pursuing further in the clinic. It is of a great advantage that one component in this combination, radiotherapy, represents an established standard of care for the majority of malignant tumors. Furthermore, radiotherapy is now also recognized as a powerful weapon for in situ vaccination, triggering a wide range of immunogenic modulations [52]. In clinical settings, the combined use of radiotherapy and cancer PDT vaccine could be preceded by a minor initial surgery for obtaining tumor material needed for preparing the PDT vaccine. An immunomodulatory treatment incorporated into the protocol will have to be in place to prevent potential immune adverse effects of surgery [75]. This should be followed by a standard radiotherapy and the PDT vaccine injection to come next immediately thereafter (with possibly additional vaccination later). Timing the use of various components will be a critical parameter to consider in the upcoming clinical trials.

## Figures and Tables

**Table 1:** PDT-generated cancer vaccines: pre-clinical studies with mouse tumor models.

Tumor Models	Vaccine Format	Reference
Mammary; mastocytoma	Cell lysates	Gollnick et al., 2002 [30]
Head and neck carcinoma	Whole cell suspension	Korbelik et al., 2006&2007 [34,35]
Glioma	Dendritic cells	Garg et al., 2016 [40]
Lung cancer	Dendritic cells	Zheng et al., 2016 [38]
Liver cancer	Supernatants of PDT-treated cells	Zhang et al., 2008 [41]
Mesothelioma	Cell lysates	Friedberg, 2006 [42]
Cervical cancer	Cell lysates	Bae et al., 2007 [43]

## References

[1] Song Q., Zhang C.-D.A., Wu X.-H. (2018). Therapeutic cancer vaccines: From initial findings to prospects. Immunol. Lett..

[2] Saxena M., van der Berg S., Melief C.J.M., Bhardwaj N. (2021). Therapeutic cancer vaccines. Nat. Rev. Cancer.

[3] ClinicalTrials.gov. https://clinicaltrials.gov.

[4] Sellars M.C., Wu C.J., Fritsch E.F. (2022). Cancer vaccines: Building a bridge over troubled waters. Cell.

[5] Cuzzubbo S., Mangsbo S., Nagarajan D., Habra K., Pockley A.G., McArdle S.E.B. (2021). Cancer vaccines; Adjuvant potency, importance of Age, Lifestyle, and treatments. Front. Immunol..

[6] Jensen K.J., Benn C.S., van Crevel R. (2016). Unraveling the nature of non-specific effects of vaccines—a challenge for innate immunologists. Semin. Immunol..

[7] Thomas S., Prendergast G.C. (2016). Cancer vaccines: A brief overview. Methods Mol. Biol..

[8] Hammerich L., Bhardwaj N., Kohrt H.E., Brody J.D. (2016). In situ vaccination for the treatment of cancer. Immunotherapy.

[9] Zhang F., Zheng Z., Barman A.K., Wang Z., Wang L., Zeng W., Wang L., Qin Y., Pandey A., Zhang C. (2021). Optimal combination treatment regimens of vaccine and radiotherapy augment tumor-bearing host immunity. Commun. Biol..

[10] Agostinis P., Berg K., Cengel K.A., Foster T.H., Girotti A.W., Gollnick S.O., Hahn S.M., Hamblin M.R., Juzeniene A., Kessel D. (2011). Photodynamic therapy of cancer: An update. CA Cancer J. Clin..

[11] Hernandez I.B., Yu Y., Ossendorp F., Korbelik M., Oliveira S. (2020). Preclinical and clinical evidence of immune responses triggered in oncologic photodynamic therapy: Clinical recommendations. J. Clin. Med..

[12] Khanam H., Dar A.M., Dar B.A., Shamsuzzaman M. (2019). Photodynamic therapy in the treatment of cancer: A review. J. Integ. Med..

[13] Dougherty T.J., Gomer C.J., Henderson B.W., Jori G., Kessel D., Korbelik M., Moan J., Peng Q. (1998). Photodynamic therapy. J. Natl. Cancer Inst..

[14] Knavel E.M., Brace C.L. (2013). Tumor ablation: Common modalities and general practices. Tech. Vasc. Interr. Radiol..

[15] Keisari Y. (2017). Tumor abolition and antitumor immunostimulation by physico-chemical tumor ablation. Front. Biosci. Landmark.

[16] Mansur A., Garg T., Shrigiriwar A., Etazadi V., Georgiades C., Habibollahi P., Huber T.C., Camacho J.C., Nour S.G., Sag A.A. (2022). Image-guided percutaneous ablation for primary and metastatic tumors. Diagnostics.

[17] Castano A.P., Demidova T.N., Hamblin M.R. (2005). Mechanisms in photodynamic therapy: Part three—Photosensitizer Pharmacokinetics, biodistribution, tumor localization and modes of tumor destruction. Photodiagnosis Photodyn. Ther..

[18] Korbelik M. (1996). Induction of tumor immunity by photodynamic therapy. J. Clin. Laser Med. Surg..

[19] Castano A.P., Mroz P., Hamblin M.R. (2006). Photodynamic therapy and anti-tumor immunity. Nat. Rev. Cancer.

[20] Gomer C.J., Luna M., Ferrario A., Wong S., Fisher A.M.R. (1996). Cellular targets and molecular responses associated with photodynamic therapy. J. Clin. Laser Surg. Med..

[21] Moserova I., Kralova J. (2012). Role of ER stress in photodynamic therapy: ROS generated in different subcellular compartments trigger diverse cell death pathways. PLoS ONE.

[22] Korbelik M. (2018). Role of stress signaling networks in cancer cell death and antitumor immune response following proteotoxic injury inflicted by photodynamic therapy. Lasers Surg. Med..

[23] Korbelik M. (2022). The outcome of tumor ablation therapies is determined by stress signaling networks. J. Cell Signal.

[24] Diaz-Villanueva J.F., Diaz-Molina R., Garcia-Gonzales V. (2015). Protein folding and mechanisms of proteostasis. Int. J. Mol. Sci..

[25] Hotamisligil G.S., Davis R.J. (2016). Cell signaling and stress responses. Cold Spring Harb. Perspect. Biol..

[26] Janssens S., Pulendran B., Lambrecht B.N. (2014). Emerging functions of the unfolded protein response in immunity. Nat. Immunol..

[27] Korbelik M., Hode T., Lam S.S.K., Chen W.R. (2021). Novel immune stimulant amplifies direct tumoricidal effect of cancer ablation therapies and their systemic antitumor immune efficacy. Cells.

[28] Lu P.D., Harding H.P., Ron D. (2004). Translation reinitiation at alternative open reading frames regulates gene expression in an integrated stress response. J. Cell Biol..

[29] Starck S.R., Shastri N. (2016). Nowhere to hide: Unconventional translation yields cryptic peptides for immune surveillance. Immunol. Rev..

[30] Gollnick S.O., Vaughan L.A., Henderson B.W. (2002). Generation of effective anti-tumor vaccines using photodynamic therapy. Cancer Res..

[31] Korbelik M., Cecic I., Nalwa H.S. (2003). Mechanism of tumor destruction by photodynamic therapy. Handbook of Photochemistry and Photobiology.

[32] Korbelik M. (2015). Impact of cell death manipulation on the efficacy of photodynamic therapy-generated cancer vaccines. World J. Immunol..

[33] Korbelik M. (2011). Cancer vaccines generated by photodynamic therapy. Photochem. Photobiol. Sci..

[34] Korbelik M., Sun J. (2005). Photodynamic therapy-generated vaccine for cancer therapy. Cancer Immunol. Immunother..

[35] Korbelik M., Stott B., Sun J. (2007). Photodynamic therapy-generated vaccine: Relevance of tumor cell death epression. Br. J. Cancer.

[36] Korbelik M., Merchant S. (2012). Photodynamic therapy-generated vaccine elicits acute phase and hormonal response in treated mice. Cancer Immunol. Immunother..

[37] Korbelik M. (2011). Optimization of whole tumor cell vaccines by interaction with phagocytic receptors. Vaccines.

[38] Zheng Y., Yin G., Le V., Zhang A., Chen S., Liang X., Liu J. (2016). Photodynamic therapy activates immune response by disrupting immunity homeostasis of tumor cells, which generates vaccine for cancer therapy. Int. J. Biol. Sci..

[39] Jung N.C., Kim H.J., Kang M.S., Lee J.H., Song J.Y., Seao H.G., Bae Y.S., Lim D.S. (2012). Photodynamic therapy-mediated DC immunotherapy is highly effective for the inhibition of established solid tumors. Cancer Lett..

[40] Garg A.D., Vandenberk L., Koks C., Verschuere T., Boon L., Van Gool S.W., Agostinis P. (2016). Dendritic cell vaccines based on immunogenic cell death elicit danger signals and T cell-driven rejection of high grade glioma. Sci. Transl. Med..

[41] Zhang H., Ma W., Li Y. (2008). Generation of effective vaccines against liver cancer by using photodynamic therapy. Lasers Med. Sci..

[42] Friedberg J. A photodynamic therapy generated tumor vaccine in an orthotopic murine malignant mesothelioma model. Proceedings of the 8th International Conference of the Mesothelioma Interest Group.

[43] Bae S.-M., Kim Y.-W., Kwak S.-Y., Kim Y.-W., Ro D.-Y., Shin J.-C., Park C.-H., Han S.-J., Oh C.-H., Kim C.-K. (2007). Photodynamic therapy-generated tumor cell lysates with CpG-oligodeoxynucleotide enhance immunotherapy efficacy in human papillomavirus 16 (E6/E7) immortalized tumor cells. Cancer Sci..

[44] Korbelik M. (2018). Immunoregulatory cell populations obligatory targets with most cancer therapies. Austin J. Clin. Med..

[45] Korbelik M., Banáth J., Saw K.M. (2015). Immunoregulatory cell depletion improves the efficacy of photodynamic therapy-generated cancer vaccines. Int. J. Mol. Sci..

[46] Korbelik M., Banáth J., Zhang W. (2016). Mreg activity in tumor response to photodynamic therapy and photodynamic therapy-generated cancer vaccines. Cancers.

[47] Korbelik M., Banáth J., Zhang W., Saw K.M., Szulc Z.M., Bielawska A., Separovic D. (2016). Interaction of acid ceramidase inhibitor LCL521 with tumor response to photodynamic therapy and photodynamic therapy-generated vaccine. Int. J. Cancer.

[48] Korbelik M., Banáth J., Zhang W., Gallagher P., Hode T., Lam S.S.K., Chen W.R. (2019). N-dihydrogalactochitosan as immune and direct antitumor agent amplifying the effects of photodynamic therapy and photodynamic therapy-generated vaccines. Int. Immunopharmacol..

[49] Klebanoff C.A., Acquavella N., Yu Z., Restifo N.P. (2011). Therapeutic cancer vaccines: Are we there yet?. Immunol. Rev..

[50] Van der Burg S.H., Arens R., Ossendorp F., van Hall T., Melief C.J. (2016). Vaccines for established cancer: Overcoming the challenges posed by immune evasion. Nat. Rev. Cancer.

[51] Gatti-Mays M.E., Redman J.M., Collins J.M., Bilusic M. (2017). Cancer vaccines: Enhanced immunogenic modulation through therapeutic combinations. Hum. Vaccin. Immunother..

[52] Cadena A., Cushman T.R., Anderson C., Barsoumian H.B., Welsh J.W., Cortez M.A. (2018). Radiation and anti-cancer vaccines: A winning combination. Vaccines.

[53] Formenti S.C., Demaria S. (2013). Combining radiotherapy and cancer immunotherapy: A paradigm shift. J. Natl. Cancer Inst..

[54] Tisch M., Heimlich F., Daniel V., Opelz G., Maier H. (1998). Cellular immune defect caused by postsurgical radiation therapy in patients with head and neck cancer. Otolaryngol. Head Neck Surg..

[55] Filatenkov A., Baker J., Mueller A.M., Kenkel J., Ahn G.O., Dutt S., Zhang N., Kohrt H., Jensen K., Dejbakhsh-Jones S. (2015). Ablative tumor radiation can change tumor immune cell microenvironment to induce durable complete remissions. Clin. Cancer Res..

[56] Belka C., Ottinger H., Kreuzfelder E., Weinmann M., Lindemann M., Lepple-Weinhues A., Budach W., Grosse-Wilde H., Bamberg M. (1999). Impact of localized radiotherapy on blood immune cells counts and function in humans. Radiother. Oncol..

[57] Vanpouille-Box C., Diamond J.M., Pilones K.A., Zavadil J., Babb J.S., Formenti S.C., Barcellos-Hoff M.H., Demaria S. (2015). Tgfbeta is a master regulator of radiation therapy-induced antitumor immunity. Cancer Res..

[58] Wennerberg E., Lhuillier C., Vanpouille-Box C., Pilones K.A., Garcia-Martinez E., Rudqvist N.P., Formenti S.C., Demaria S. (2017). Barriers to radiation-induced in situ tumor vaccination. Front. Immunol..

[59] Ma Y., Kepp O., Ghiringhelli F., Apetoh L., Aymeric L., Locher C., Tesniere A., Martins I., Ly A., Haynes N.M. (2010). Chemotherapy and radiotherapy: Cryptic anticancer vaccines. Semin. Immunol..

[60] Chakraborty M., Abrams S.I., Camphausen K., Liu K., Scott T., Coleman C.N., Hodge J.W. (2003). Irradiation of tumor cells up-regulates Fas and enhances CTL lytic activity and CTL adoptive immunotherapy. J. Immunol..

[61] Garnett C.T., Palena C., Chakraborty M., Tsang K.Y., Schlom J., Hodge J.W. (2004). Sublethal irradiation of human tumor cells modulates phenotype resulting in enhanced killing by cytotoxic T lymphocytes. Cancer Res..

[62] Gameiro S.R., Jammeh M.L., Wattenberg M.M., Tsang K.Y., Ferrone S., Hodge J.W. (2014). Radiation-induced immunogenic modulation of tumor enhances antigen processing and calreticulin exposure, resulting in Enhanced T-cell killing. Oncotarget.

[63] Vu M.D., Xiao X., Gao W., Degauque N., Chen M., Kroemer A., Killeen N., Ishii N., Li X.C. (2007). OX40 costimulation turns off Foxp3+ Tregs. Blood.

[64] Garnett-Benson C., Hodge J.W., Gameiro S.R. (2015). Combination regimens of radiation therapy and therapeutic cancer vaccines: Mechanisms and opportunities. Semin. Radiat. Oncol..

[65] Kumari A., Cacan E., Greer S., Garnett-Benson C. (2013). Turning T cells on: Epigenetically enhanced expression of effector T-cell costimulatory molecules on irradiated human tumor cells. J. Immunother. Cancer.

[66] Matsumura S., Wang B., Kawashima N., Braunstein S., Badura M., Cameron T.O., Babb J.S., Schneider R.J., Formenti S.C., Dustin M.L. (2008). Radiation-induced CXCL16 release by breast cancer cells attracts effector T cells. J. Immunol..

[67] Huang Y., Goel S., Duda D.G., Fukumura D., Jain R.K. (2013). Vascular normalization as an emerging strategy to enhance cancer immunotherapy. Cancer Res..

[68] Deng L., Liang H., Burnette B., Beckett M., Darga T., Weichselbaum R.R., Fu Y.X. (2014). Irradiation and anti-PD-L1 treatment synergistically promote antitumor immunity in mice. J. Clin. Investig..

[69] Kroemer G., Galuzzi L., Kepp O., Zitvogel L. (2013). Immunogenic cell death in cancer therapy. Annu. Rev. Immunol..

[70] Mondini M., Nizard M., Tran T., Mauge L., Loi M., Clemenson C., Dugue D., Maroun P., Louvet E., Adam J. (2015). Synergy of radiotherapy and a cancer vaccine for treatment of HPV-associated head and neck cancer. Mol. Cancer Ther..

[71] Adotevi O., Vingert B., Freyburger L., Shrikant P., Lone Y.C., Quintin-Colonna F., Quintin-Colonna F., Haicheur N., Amessou M., Herbalin A. (2007). B subunit of Shiga toxin-based vaccines synergize with alphagalactosylceramide to break tolerance against self antigen and elicit antiviral immunity. J. Immunol..

[72] Gulley J.L., Arlen P.M., Bastian A., Morin S., Marte J., Beetham P., Tsang K.-Y., Yokokawa J., Hodge J.W., Menard C. (2005). Combining a recombinant cancer vaccine with standard definitive radiotherapy in patients with localized prostate cancer. Clin. Cancer Res..

[73] Shibamoto Y., Okamoto M., Kobayashi M., Ayakawa S., Iwata H., Sugie C., Mitsuishi Y., Takahashi H. (2013). Immune-maximizing (IMAX) therapy for cancer: Combination of dendritic cell vaccine and intensity-modulated radiation. Mol. Clin. Oncol..

[74] Khurana D., Martin E.A., Kaspbauer J.L., O’Malley B.W., Salomao D.R., Chen L., Strome S.E. (2001). Characterization of a spontaneously arising murine squamous cell carcinoma (SCC VII) as a prerequisite for head and neck cancer immunotherapy. Head Neck.

[75] Bakos O., Lawson C., Rouleau S., Tai L.-H. (2018). Combining surgery and immunotherapy: Turning and immunosuppressive effect into a therapeutic opportunity. J. Immunother. Cancer.

